# Freeform Wide Field-of-View Spaceborne Imaging Telescope: From Design to Demonstrator

**DOI:** 10.3390/s22218233

**Published:** 2022-10-27

**Authors:** Luca Schifano, Michael Vervaeke, Dries Rosseel, Jef Verbaenen, Hugo Thienpont, Steven Dewitte, Francis Berghmans, Lien Smeesters

**Affiliations:** 1 Brussels Photonics (B-PHOT), Department of Applied Physics and Photonics, Vrije Universiteit Brussel, Pleinlaan 2, 1050 Brussels, Belgium; 2 Royal Meteorological Institute of Belgium, Avenue Circulaire 3, 1180 Brussels, Belgium; 3 Flanders Make, Pleinlaan 2, 1050 Brussels, Belgium; 4 Royal Observatory of Belgium, Avenue Circulaire 3, 1180 Brussels, Belgium

**Keywords:** wide field-of-view, telescope, freeform optics, reflective optics, space instrumentation, demonstrator, Earth observation, climate monitoring

## Abstract

Wide field-of-view imaging optics offer a huge potential for space-based Earth observation enabling the capture of global data. Reflective imaging telescopes are often favored, as they do not show chromatic aberrations and are less susceptible to radiation darkening than their refractive counterparts. However, the main drawback of reflective telescopes is that they are limited with respect to field-of-view while featuring large dimensions. We propose the use of freeform optics to maximize the field of view while maintaining diffraction-limited image quality and minimizing system dimensions. In this paper, we present a novel freeform wide field-of-view reflective telescope, starting from the optical design, and continuing to tolerancing analysis and manufacture, towards a proof-of-concept demonstrator. The novel telescope features a full field-of-view of 120° while showing an exceptional spatial resolution of 2.6 km and fitting within 1 CubeSat unit. To the best of our knowledge, this is the widest field-of-view that has ever been realized for a space-based telescope, nearly reaching Earth observation from limb to limb from an altitude of about 700 km. We hope this design paves the way for future space missions enabling improved Earth observation and leading to enhanced monitoring of climate and climate change.

## 1. Introduction

Earth observation from space provides an endless source of vital information for weather forecasting, sea monitoring, monitoring of forest fires and deforestation, and agriculture production cycles, to name but a few [1,2,3]. In view of climate change, Earth observation is indispensable for monitoring the effects of increasing temperatures [4,5,6]. To obtain accurate information with a high spatial resolution and temporal coverage, ongoing research and evaluation tend towards miniaturization and thus the use of cost-effective small satellites that are suitable for use in satellite constellations.

Imaging telescopes can be considered one of the key instruments for Earth observation. Reflective imaging telescopes are widely used for space applications, and often favored above refractive designs as the latter often suffer from chromatic aberrations when considering broadband imaging designs. A wide diversity of reflective telescope designs has already been proposed, of which the Three Mirror Long and WALRUS designs have been shown to present the largest field-of-view (FOV), able to reach 90° full angle, but at the cost of a large lens diameter [7]. Over the last decade, freeform optics has been shown to offer a large potential for space telescopes, enabling a decrease in the mass and volume of the optical systems, while improving the optical performance and image quality [8,9]. Freeform optical components are generally defined as optical elements whose surfaces lack translational and rotational symmetry. In contrast to spherical and aspheric optical surfaces, which are described by the radius of curvature and a limited number of aspheric coefficients, freeform surfaces are generally described as point clouds or via the use of a combination of polynomial functions, adding a high number of degrees of freedom to the design. Recent advancements in freeform technology, including design methodologies, manufacturing, and metrology capabilities, have enabled this approach to surpass classic reflective designs in terms of FOV and f-number while enabling the minimizing of the number of optical elements. A freeform mirror telescope system was integrated into the Tropospheric Monitoring Instrument (TROPOMI), developed in the framework of the ESA Copernicus space mission and launched in 2017 on the Sentinel-5 precursor satellite. The freeform components enabled the telescope to cover a high spectral range (UV to short wave infrared (SWIR) wavelengths), to reach a high resolution of 7 × 7 km² and a large FOV of 108° while correcting for geometrical aberrations [10,11]. In addition, Jahn et al. proposed a freeform three-mirror space telescope to attain high-resolution imaging for Earth or planetary science [12], while Challita et al. outlined the use of freeform mirrors in astronomical instruments [13].

We propose an innovative two-mirror pushbroom telescope imaging the Earth with 120° FOV, featuring a spatial resolution of 2.6 km while fitting in 1 CubeSat Unit and showing a diffraction-limited performance. The telescope can be used as a stand-alone component, but also serve as input for a spectroscopy unit [14]. Pushbroom non-scanning wide FOV instruments, such as the Ozone Monitoring Instrument (OMI), TROPOMI, and the future UVN and UVNS instruments, have inspired the design of our instrument. As in the TROPOMI telescope, we use two freeform mirrors to achieve the extremely wide FOV. However, in comparison with TROPOMI, our goal is to improve the spatial resolution from 7 km to 2.6 km at the nadir, while extending the FOV from 108° to 120°, almost reaching an Earth observation from limb to limb from an altitude of about 700 km. We envision the use of our instrument in the near-infrared wavelength range (1100–1700 nm). This wavelength range is complementary to TROPOMI [11] and would enable greenhouse gas monitoring when supplementing the telescope with a spectrometer. More specifically, carbon dioxide column densities have been observed at 1600 nm [15,16], while water vapor shows characteristic absorbances around 1130 nm and 1400 nm [14], and methane features a spectral line at 1670 nm [17]. Cameras and telescopes operating within the visible wavelength range (400–900 nm) are already widely available. When supplementing these visible imaging designs with the proposed telescope, a more complete view of the Earth’s total reflected radiation can also be obtained. In addition, and given the ongoing tendency towards more compact designs enabling the use of low-cost satellites, we aim for our telescope to fit within 1 CubeSat Unit. This way, in the longer term, the use of several CubeSats would allow a constellation of satellites for the same cost as a large-scale space mission, and with the benefit of a much higher scientific yield.

This paper presents our novel two-mirror freeform telescope design, starting from the optical design and proceeding to tolerancing, manufacturing, and proof-of-concept demonstration. Section 2 presents the optical design and performance evaluation, as well as a tolerance analysis. Section 3 discusses the manufacturing and proof-of-concept demonstration. Next, the results and future perspectives are discussed in Section 4. Finally, we close this paper with conclusions given in Section 5.

## 2. Methods: Optical Design

### 2.1. Optical System Design

**Figure 1:**
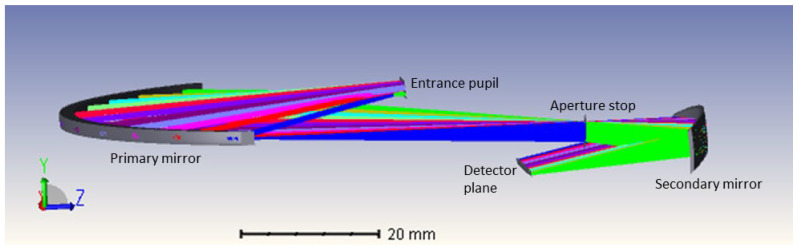
Optical system layout of the freeform two-mirror imaging telescope design. The light travels sequentially from the entrance pupil to the primary mirror, aperture stop, secondary mirror, and detector plane.

The optical design and analysis were performed using Zemax OpticsStudio^®^. The design comprises two freeform mirrors, mounted off-axis, including an aperture stop located between the two mirrors at the focal point of the secondary mirror (Figure 1). The design is optimized within the 1100 nm and 1700 nm wavelength range, for a full FOV of 120°, and features an f-number of 7.23. Each of the fields is imaged on a point, resulting in a line on the detector plane. Additionally, telecentricity is achieved at this detector plane. The design has dimensions of 96 mm × 15 mm × 95 mm, fitting within 1 CubeSat Unit.

The final design is achieved using a two-step design process. First, an on-axis telescope without tilts, but including two mirrors and the slit after the secondary mirror was considered for optimization of the freeform terms, after which the tilts were introduced to avoid vignetting, and re-optimization was performed. This two-step approach enabled the decorrelation of the shape and geometry of the mirrors, avoiding possible confusion. The freeform mirrors are described and optimized using XY polynomials, as the telescope is off-axis with respect to the Y-axis and features a symmetrical FOV with respect to the X-axis. The surface sag (z) comprises a base conic aspherical surface upon which the polynomial aspheric terms are added, as described by equation 1, in which c is the reciprocal of the radius, r is the radial coordinate in lens units, k is the conic constant, N is the number of polynomial coefficients and $A_{i}$ is the coefficient of the *i*th extended polynomial term. The polynomials $E_{i}\left(x,y\right)$ are a power series in *x* and *y*, where the position values *x* and *y* are divided by a normalization radius, implying the polynomial coefficients are dimensionless [18].

(1) $$z=\frac{c\;r^{2}}{1+\sqrt{1-\left(1+k\right)c^{2}r^{2}}}+\overset{N}{\underset{i=1}{\sum }}A_{i}E_{i}\left(x,y\right)\;$$

We optimize the shape of the freeform mirrors for 11 fields between +60° and −60°, including their radius of curvature and the following XY polynomial terms: X2Y0, X0Y2, X0Y3, X4Y0, X2Y2, X0Y4, X2Y3, X0Y5, X6Y0, and X4Y2. Since we have an orthogonal symmetry along the optical axis (i.e., +X and −X coefficients are symmetric, as are the fields +x° and −x°), we only need even orders in X, such as X0, X2, X4, and X6. Therefore, we do not consider odd orders in X, such as X1, X3, and X5. We first considered terms up to the 4th order, while after introducing the tilts in the design, we add the 5th order term X0Y5 and 6th order term X6Y0, to further correct for aberrations. An iterative optimization was performed, considering the spot size and modulation transfer function (MTF) in the merit function. The total track length, f-number, entrance pupil diameter, and the ray positions given telecentricity were constrained in the merit function to fulfill the pursued specifications.

**Figure 2:**
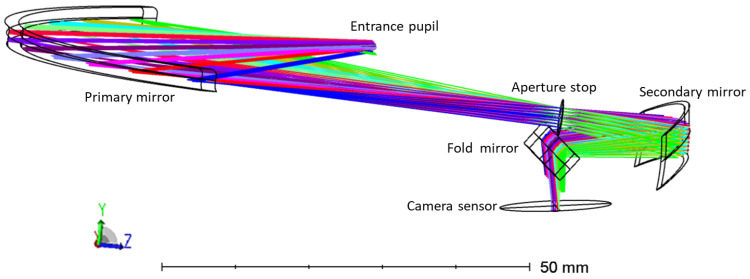
Optical design of the telescope proof-of-concept demonstrator, including a fold mirror enabling the use of a Xenics Bobcat 320 GigE 100 camera at the detector plane.

Considering the final application and integration, the freeform lenses will be mounted monolithically to ensure fixed positioning, while the imaging detector or entrance slit of the spectrometer can be included at the specified detector plane in Figure 1. However, for the purpose of our laboratory demonstrator, the optics needed to be mounted using bulky optomechanics featuring micrometer screws to enable an evaluation of the mounting constraints, while a commercial off-the-shelf (COTS) camera was used (Xenics Bobcat 320 GigE 100 camera) to assess the image quality, consuming more space (55 × 55 × 82 mm^3^) than an OEM image sensor mounted on a printed circuit board would. Taking the larger dimension of the mounting optomechanics and the camera into account, a dummy thin fold mirror is added to the design, in a way that does not affect the image quality but relaxes the space constraint on the positioning of the detector. This results in a final layout of the demonstrator setup as presented in Figure 2.

### 2.2. Performance Evaluation

**Figure 3:**
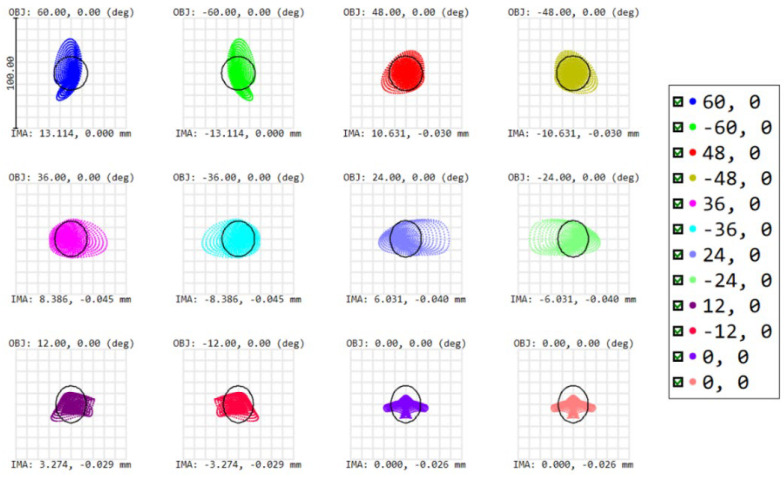
Spot diagram indicating all RMS spot sizes are approximately the size of the Airy disk (indicated by the black circle). Symmetry is properly achieved with respect to 0°. OBJ (in degrees) defines the object field and IMA (in mm) defines the image height of the centroid on the detector. The different colors correspond to the different fields.

**Figure 4:**
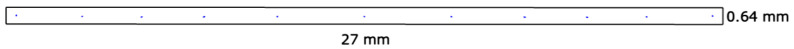
Relative position of the spots on the image plane, considering the simulated fields of Figure 3.

**Figure 5:**
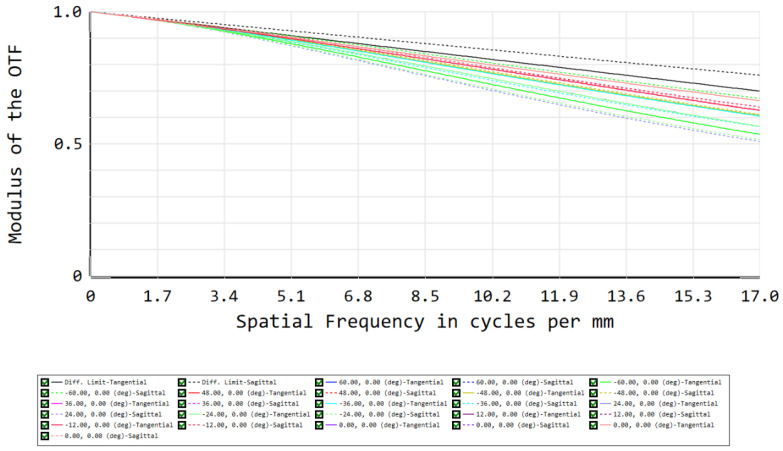
MTF evaluation of the telescope, where the different colors correspond to the different fields shown in Figure 3 and the black line corresponds to the diffraction limit. The full and dashed lines correspond to the tangential and sagittal planes, respectively.

The simulated image quality is evaluated using the root-mean-square (RMS) spot size and MTF. Considering the RMS spot diagram, the spot sizes for each of the fields approximate the Airy disk radius that equals 15 µm, indicating a diffraction-limited performance (Figure 3). All presented spots are located next to each other on the image sensor. The relative positioning of the spots with respect to each other on the detector plane is visualized in Figure 4, indicating the spots are focused on a line, showing only minor spatial distortion induced by the present barrel distortion. The barrel distortion is the main impacting aberration on the performance, increasing with the field angle and reaching a maximum value of 23% at 60°, but can be corrected using post-processing. As a second evaluation, the performance is confirmed by the MTF, showing a close to diffraction-limited design for all fields and both tangential and sagittal rays (Figure 5). MTF values between 0.5 and 0.7 are obtained for all fields at 17 cycles/mm, while the diffraction limit at this frequency gives a value of about 0.75.

### 2.3. Tolerance Analysis

To evaluate the required manufacturing and alignment accuracy, tolerance analysis is performed on the optical telescope design. This analysis contains two types of tolerances: (1) surface tolerances including manufacturing defects of the shapes of the two freeform mirrors (Table 1), and (2) element tolerances, including possible misalignments of the mirrors, stop and detector plane (Table 2). The evaluation is performed at a wavelength of 1.7 µm, the upper limit of the considered spectral range, and using focus compensation.

The tolerance analysis is performed using a statistical Monte Carlo analysis considering 1000 iterations on the optical design while aiming to evaluate if the decrease in optical performance is still acceptable when taking the (surface and element) tolerances into account. For this, the RMS spot sizes and MTF were considered figures of merit during the evaluation. A worst-case evaluation indicates that the mean (averaged over all fields) RMS spot value equals less than 11.3 µm at the detector plane and that the mean MTF exceeds 0.44 at 17 cycles/mm, for more than 98% of the traceable Monte Carlo files. More than 80% of our simulated designs show less than a 15% deviation for the RMS spot size and MTF. As a result, this tolerance analysis shows a robust optical design, giving us high confidence to achieve a tolerant and efficient telescope.

## 3. Telescope Proof-of-Concept Demonstrator

### 3.1. Manufacturing and Alignment Process

**Figure 6:**
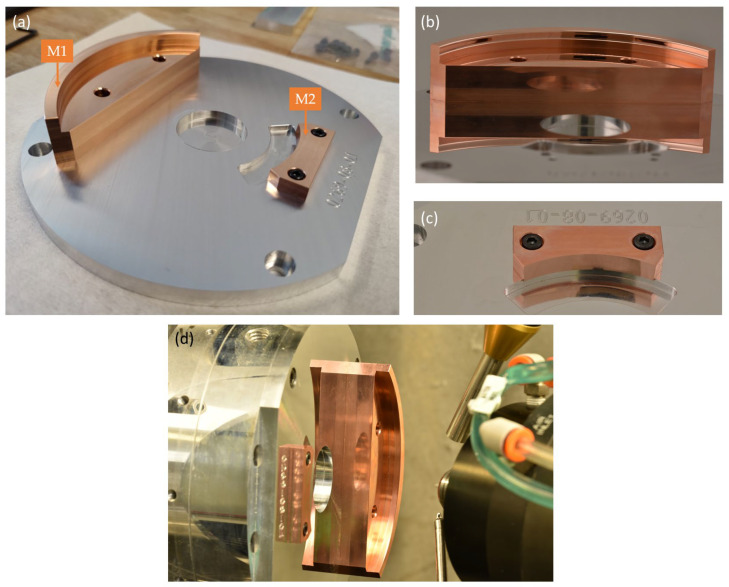
Manufacturing of the freeform telescope mirrors: (**a**) both mirrors positioned on the mounting structure, after polishing and before ultraprecision diamond tooling. M1 is the primary mirror and M2 the secondary mirror; (**b**) close-up of the primary mirror; (**c**) close-up of the secondary mirror; (**d**) diamond tooling generating the freeform surface shape and optical surface quality.

The manufacturing of freeform mirrors requires high-precision multi-axis milling, based on computer-aided design (CAD). The two mirrors are made of oxygen-free high thermal conductivity (OFHC) copper, which is the material of choice for broadband infrared applications [19,20]. Starting from two flat OFHC copper blanks, a preliminary shape is first generated using high-precision 5-axis milling (Röders TEC), as presented in Figure 6a. Next, 5-axis ultraprecision diamond tooling (Nanotech 350FG) is used to superimpose the freeform terms on the two mirror surfaces, while ensuring high optical surface quality (Figure 6d). This latter process takes about 30 h, for a high precision (accuracy of 10 µm) of the XY polynomial freeform coefficients. The combination of both manufacturing devices enables the accurate manufacturing of the freeform surface shapes (Figure 6b,c). However, precise positioning and alignment of the mirrors with respect to each other, and with the milling tool on both manufacturing devices, is also indispensable. To ensure that minimal manufacturing errors are introduced when mounting the mirrors from the milling tool to the diamond tooling device, both copper parts were first mounted on a single mounting plate, after which the milling process took place. From the start of the manufacturing process, both copper blanks were thus monolithically mounted in a fixed position. The mounting plate was generated using the Röders 5-axis milling tool ensuring a precise diameter (runout of 8 µm with respect to the XY origin) and including a reference plate. Additionally, four alignment pins were used to enable accurate positioning of the copper parts. To ensure the same alignment during the milling process as during the diamond tooling, the same references were used during the alignment. The diamond milling tool could be aligned with an accuracy of 0.5 µm with respect to the rotation center. The manufacturing of the mirrors was fully realized in-house, at the VUB B-PHOT Brussels Photonics Innovation Centre.

**Figure 7:**
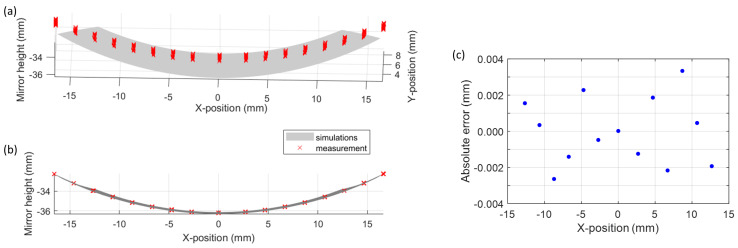
Validation of the manufactured surface shape using CMM: (**a**) 3D overview of the measurements, where the measured surface profile is indicated in red and the simulated one in grey; (**b**) cross-section along the mirror surface, enabling a comparison between the measured and simulated profile; and (**c**) absolute error achieved by subtracting the measured and simulated mirror profiles.

Validation of the manufacturing process was performed on the secondary mirror by a characterization of the surface shape and roughness, using in-house metrology tools in a cleanroom environment. Surface roughness of 10 nm was measured using a white-light interferometer, considering a field-of-view of 0.17 mm × 0.22 mm and a 2nd-order Gaussian regression filter. Using a coordinate measurement machine (CMM), the surface shape of the mirror was validated, indicating a maximum error of ±3.3 µm with respect to the simulated mirror surface (Figure 7). The surface of the primary mirror could not be reached due to the monolithic mounting but is expected to show a similar performance as the same cutting parameters were applied to both surfaces.

As a last step during the manufacturing process, the aperture stop and a flat fold mirror are added. The additional mirror enables the mounting of the Xenics Bobcat 320 GigE 100 Hz camera, and is mounted on a movable structure, ensuring coverage of the full FOV.

### 3.2. Laboratory Demonstrator Setup

**Figure 8:**
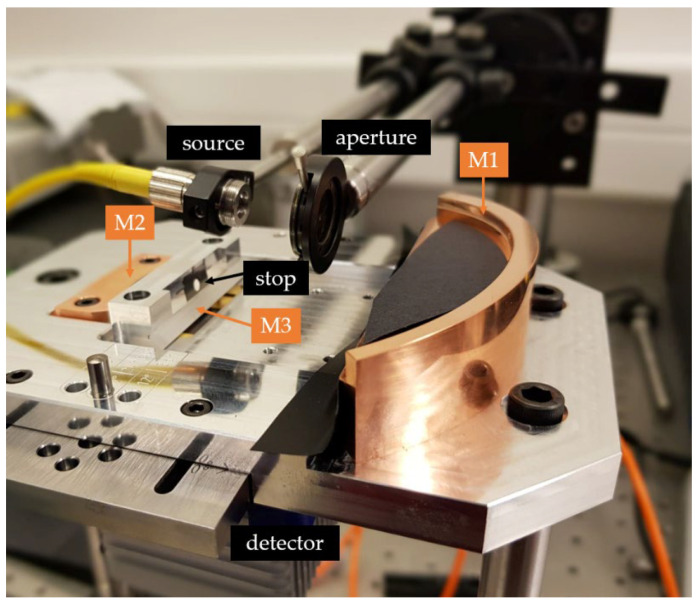
Experimental proof-of-concept demonstrator. After reflection on M1, light passes through the aperture stop and is reflected by the secondary (M2) and fold mirror (M3) to the detector that is located beneath the telescope.

The manufactured telescope is combined with an illumination source, aperture, and detector to validate its imaging performance (Figure 8). The incoming light is reflected on the primary mirror (M1), directing the light to the aperture stop and the secondary mirror (M2), which reflects the light to the fold mirror (M3) that guides the light to the detector, which is located beneath the telescope. For this, a three-step approach was followed during the laboratory demonstrator measurements: (1) alignment of the full setup, including source, detector, and aperture, using a collimated visible red laser source, (2) performance evaluation and spot-size measurement when replacing the red laser source with a near-infrared (NIR) laser emitting light within the envisioned wavelength range (1100–1700 nm), and (3) stray light evaluation using the red laser source with the aid of backward ray tracing. During each of the three steps, a collimated input beam is used, followed by the aperture, both mounted on translation and rotation stages, enabling the scanning of the performance along the FOV (120°). Preference is given to fiber-pigtailed sources to ease the mounting. Each of the three steps is explained in detail in the following subsections.

#### 3.2.1. Telescope Alignment Using Red Laser Source

A fiber-pigtailed red laser source (Thorlabs S1FC635, emission wavelength of 635 nm, 2.5 mW output power, single mode emission) is used for alignment purposes, supplemented with a collimating lens to achieve a collimated input beam (beam diameter 891 µm). Using this laser source, we sought to accurately position the source and aperture in alignment with the primary mirror, such that the light passes through the aperture stop (Figure 8). The detector position is fixed below the fold mirror. The alignment and tilt of the laser source are set such that an angle of incidence of zero degrees results in a spot at the center of the image sensor.

#### 3.2.2. Performance Validation Using NIR Laser Source

The imaging performance is evaluated using a near-infrared laser source at 1550 nm, at the center of the spectral range that is targeted with this telescope (1100–1700 nm), and within the sensitive operation region of the Xenics sensor (900–1700 nm). For this, an APEX AP2080C tunable laser source was used and set to an output wavelength of 1550 nm and optical output power of 339.63 µW. An optical fiber is connected to the light output unit, to guide the light to the telescope, supplemented with a collimating lens to achieve a collimated input beam. The Xenics Bobcat 320 GigE 100 was used as a SWIR camera, featuring an InGaAs sensor with a 320 × 256-pixel resolution and a pixel pitch of 20 µm.

**Figure 9:**
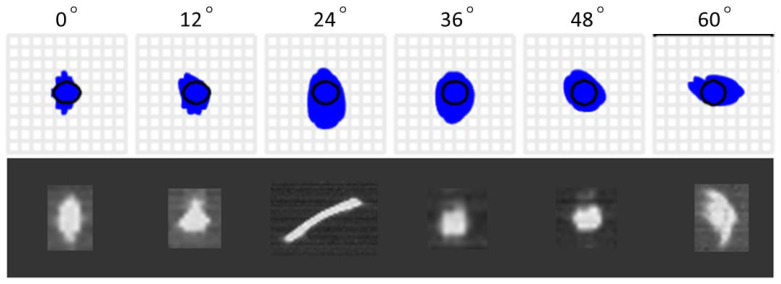
Simulations versus measurements at the detector. The top row shows the simulated spots for different field angles, while the bottom row presents the measured spots. The measurements validate the simulations, except for ±24°, where we suspect stray light caused by a manufacturing error.

**Figure 10:**
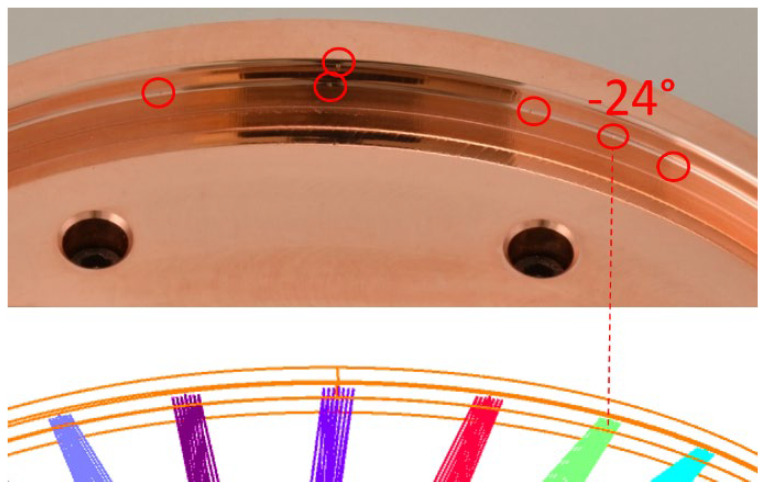
Small defects, highlighted by red circles, can be observed in the primary mirror and the mounting structure. One is particularly important because it is located exactly at the −24° field, as indicated by the bottom field plot, probably causing the stray light that is observed for this field. The different colors in the bottom figure correspond to the different fields.

A full FOV of 120° was successfully observed. The measured spot sizes, at different field angles, show a good consistency with the simulated spot sizes (Figure 9). Note that we observe geometric spots and not the RMS spot sizes. Nevertheless, the spot sizes are of the same order of magnitude as when considering the simulations executed in the sequential mode. The shape of the spots also shows a good resemblance. A deviating spot arises at ±24°, where we observe stray light reaching the detector. We suspect this stray light arises from a manufacturing error, as a small defect near the primary mirror is present at this field (Figure 10). An accurate inspection of the mirror surface indicated some minor defects just at the edge of the mirror area, induced by small overshoots at the lead-in and lead-out positions of the diamond tool. In theory, these points are outside of the mirror surface and thus not illuminated in the simulations. However, since the RMS spot size is considered during the simulations, the tails of the Gaussian input beam might reach these minor defect points in the demonstrator, inducing light reflection and scattering.

**Figure 11:**
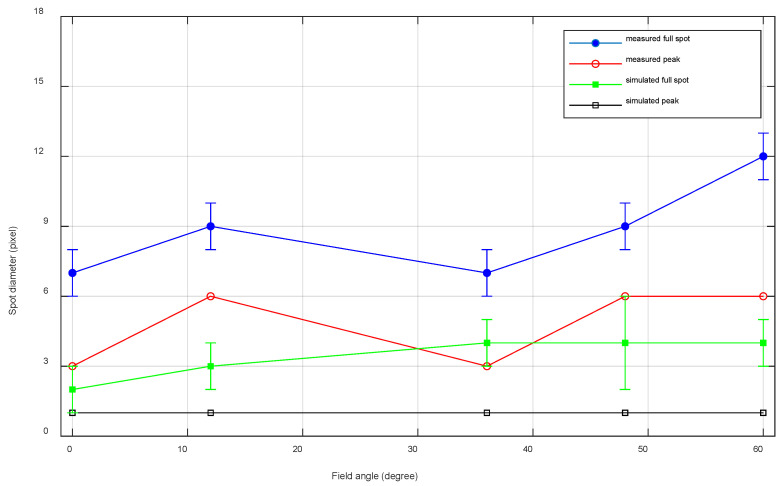
Comparison between the number of pixels (pixel pitch = 20 µm) needed to image the simulated spots and the number of pixels on which the spots were imaged using the laboratory demonstrator. For each field angle, the peak intensity of each spot (peak), as well as the full spot is considered. Error bars are given on the full spots. The error on each measured full spot is 1 pixel, while the error bars on the simulated spots are given by the estimated changes (dependent on each field) in the tolerance analysis.

Next, a more quantitative comparison is performed by evaluating the simulated and measured spot diameter. By rounding the simulated spot sizes to the number of pixels needed to image them, we enable a comparison with the illuminated pixels and thus spot sizes using the laboratory demonstrator (Figure 11). The measured and simulated spots show a similar size along the full FOV. For both the measurements and the simulations, we consider both the full spot diameter and the peak intensity. The simulated full spot diameter shows a good match with the measured peak intensity. However, the measured full spot diameters are consistently giving slightly larger values. Several factors might affect these latter spot sizes, such as stray light, possible manufacturing defects, or too large tolerances coupled with the absence of a compensator, as the distance between the fold mirror and the detector is fixed and imposed by the mounting plate. Additional simulations indicated the high importance of this compensator as minor detector movements strongly impact the spot size on the detector surface.

#### 3.2.3. Stray Light Analysis

While we qualitatively demonstrated the proof-of-concept of our telescope, small deviations in the performance were observed, mainly originating from stray light that is induced by the tilt of the primary mirror and the manufacturing defects at the edges of the mirrors. To gain more insight into the stray light and its origin, an in-depth evaluation was performed using red laser light, replacing the visual observation of the stray light within the laboratory setup. For this, we used a collimated red laser (780 nm, beam diameter of 850 µm), in combination with the Gentec BEAMAGE-4M beam profiling camera as the detector.

**Figure 12:**
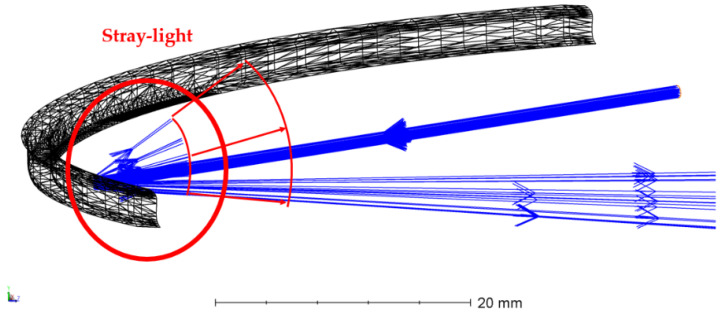
Stray light induced by a minor misalignment of the source and primary mirror, simulated within the non-sequential mode. When the source is not properly tilted around X, stray light occurs after reflection on the primary mirror, causing a vertical beam.

The tilt of the primary mirror with respect to the laser source appeared to be a highly sensitive and important parameter influencing the stray light. Despite the presence of a black absorbing sheet to reduce the amount of stray light, a suboptimal tilt of the light source with respect to the primary mirror induces an elliptical vertical beam (Figure 12). In the current setup, and because of the monolithically mounting, the tolerance on this tilt could not be accurately determined. However, the presence of stray light was cross-checked with the simulations using Zemax OpticsStudio non-sequential mode, indicating a vertical beam when the source is not properly tilted around X, thus confirming these laboratory observations.

**Figure 13:**
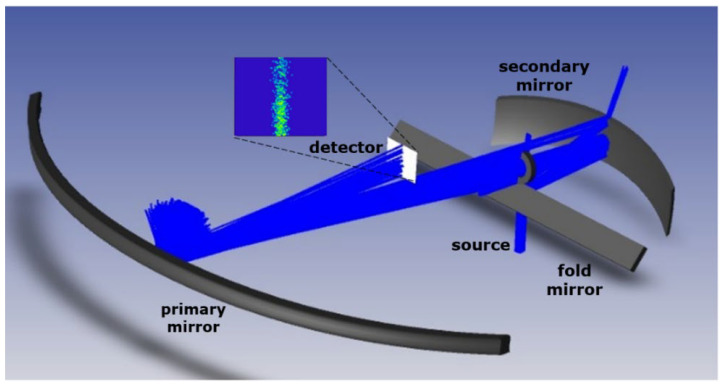
Backward ray tracing from the fold mirror to the secondary mirror and primary mirror. The detector is located at the position of the former entrance pupil. An elliptical vertical spot is obtained at the detector.

**Figure 14:**
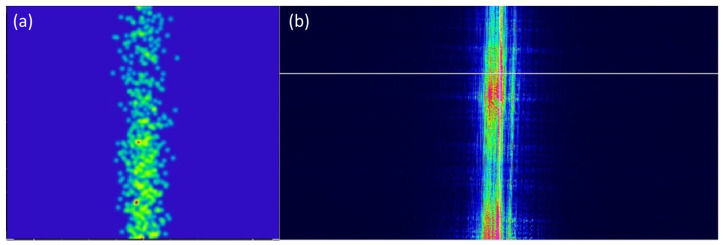
Scattered stray light beam evaluation when considering backward ray-tracing and illumination: (**a**) simulation result, (**b**) spot measurement, where the color corresponds to the beam intensity distribution. Both simulations and measurements show a similar performance.

An additional evaluation was performed using backward illumination, by interchanging the source and detector. This evaluation enables us to focus on the stray light presence while excluding the potential positioning error on the detector, which was also used as a compensator during the tolerance analysis. To enable a cross-check between the simulations and measurements, the ray-tracing simulations are adapted according to the backward laboratory configuration, using the non-sequential mode (Figure 13). For this, the former detector is replaced by the collimated laser source, and a new detector is inserted at the position of the entrance aperture. During both the simulations and measurements, a vertical line is achieved on the detector (Figure 14). As the incident beam size slightly exceeds the size of the secondary mirror, this line signal is induced by scattering on the edges inducing stray light in the vertical direction. The thickness of the vertical beam approximately equals 1300 µm for the simulation and 1815 µm for the measurements. The measurements show a slightly larger beam due to the imperfections on the sides of the mirror as indicated in Figure 10.

## 4. Discussion and Future Perspectives

### 4.1. Short-Term Perspectives and Comparison with the State-of-the-Art

Pushbroom non-scanning wide FOV instruments, such as OMI, TROPOMI, and the future UVN and UVNS instruments, have inspired the design of our telescope. As with the TROPOMI telescope, we use two freeform mirrors featuring an extremely wide FOV [11]. However, unlike TROPOMI, which shows a FOV of approximately 108°, our telescope features a FOV of 120°, which is the widest field angle that has ever been achieved for a space-based telescope. Our extremely wide FOV nearly allows Earth observation from limb to limb from an altitude of about 700 km. On top of that, our spatial resolution equals 2.6 km × 2.6 km, which is about 2.7 times better than TROPOMI. Consequently, our design not only surpasses previous instruments in terms of FOV but also in terms of spatial resolution, all while fitting within 1 CubeSat Unit.

The operation of our novel freeform telescope is evaluated within a first qualitative proof-of-concept demonstrator setup. The realization of this proof-of-concept demonstrator involved tackling advanced freeform manufacturing and precise mounting of the mirror surfaces. In general, a similar optical performance is observed for the simulations and measurements, validating the imaging performance and potential of our future space-based telescope. Possible further improvements might be achievable by a re-evaluation of the mounting structure of the mirrors. More specifically, we believe the performance might benefit from the following five improvements: (1) adaptation of the lead-in and lead-out positions of the diamond tool, avoiding overshoot of the machining causing defects close to the optical surface; (2) use of a black coating on the structure holding the mirrors to reduce stray light; (3) use of a physical aperture located at the entrance pupil, in combination with a baffle located at the entrance of the system, to ease the alignment and reduce stray light; (4) adding an adaptable tilt to the mirrors, especially for the primary mirror, as the angle between the light source and the primary mirror appears to be critical; (5) adaptation of the detector positioning tools inducing additional degrees of freedom allowing compensation for tolerances, by using the distance between the third mirror and the detector as a compensator.

Future research will also include a detailed analysis of the radiometric budget. A back-to-the-envelope calculation, in combination with a comparison with the TROPOMI telescope, gives confidence that a sufficient signal-to-noise ratio (SNR) can be achieved. Our novel telescope features a small entrance pupil diameter (2.52 mm for our instrument vs. 3 mm for TROPOMI), targets an excellent spatial resolution (2.6 km vs. 7 km for TROPOMI), and thus a short maximum integration time (371 ms for a spatial resolution of 2.6 km at nadir vs. 1 s for 7 km for TROPOMI), which tend to decrease the SNR. However, this is balanced by the wider FOV (120° vs. 108°) and smaller f-number (7.23 vs. 9–10), which tend to increase the SNR.

### 4.2. Long-Term Perspectives towards Future Space Missions

As a possible application, the pushbroom telescope might be complemented with a spectrometer unit, for example, for climate change and/or pollution monitoring [14]. Recently, the need for compact spectrometers for enhanced climate and pollution monitoring has been put forward, as highlighted by, for example, the CHAPS instrument that targets the monitoring of local pollution in urban areas [21]. Possible integration with an 1100–1700 nm spectrometer might enable greenhouse gas monitoring [14,15,16,17]. Also, as our telescope achieves an RMS spot diameter of the order of 30 µm or less at the detector plane, this telescope would be compatible with other spectrometers operating within wavelength ranges exceeding 1700 nm, while keeping a diffraction-limited design for the telescope. In particular, the thermal infrared region (8–14 µm) could be of interest for the monitoring of trace gases in the atmosphere, while highlighting the direct link between greenhouse gases and the Earth’s outgoing longwave radiation.

From a long-term perspective, we foresee launching the proposed telescope on board a small satellite at a Low-Earth-Orbit altitude of 700 km. The satellite could, for example, be a CubeSat companion to the Earth Climate Observatory (ECO) pursuing improved radiation monitoring at the top-of-atmosphere [22,23]; while, alternatively, a SmallSat platform (such as a PROBA satellite) can be considered. The use of CubeSat or SmallSat satellite platforms holds promise for a faster development time at a reduced cost, implying that, for the same cost as a large space mission, multiple copies of this satellite, i.e., a constellation of satellites, can be realized leading to improved monitoring of our planet.

## 5. Conclusions

This paper presents a novel space-based Earth-observing telescope, starting from the optical design, and proceeding to tolerancing, manufacturing, and proof-of-concept demonstration. Our novel telescope is composed of two freeform mirrors. Freeform optics allows a close to diffraction-limited image quality to be achieved, whilst minimizing the number of mirrors, maximizing the field-of-view, and minimizing the system dimensions. Featuring a field-of-view of 120°, we achieve the widest field-of-view that has ever been realized for a space-based telescope, enabling Earth observation nearly from limb to limb from an altitude of about 700 km. In addition, our spatial resolution of 2.6 km × 2.6 km exceeds the state-of-the-art, while the design of the full instrument is compact, fitting within 1 CubeSat Unit. We believe the proposed telescope might be the candidate of choice for future space missions that target wide field-of-view imaging and/or enhanced monitoring of climate change.

## Figures and Tables

**Table 1:** Surface tolerances using the *High Precision* tolerances from Edmund Optics. Note that we use *precision* for the Radius, as *high precision* would mean 0%, which would be unfeasible.

	Surface Tolerances
Radius	0.1% (precision)
Thickness	0.01 mm
Decenter X	0.01 mm
Decenter Y	0.01 mm
Tilt X	0.0167°
Tilt Y	0.0167°
Irregularity	0.2 fringes

**Table 2:** Element tolerances that can be realized using COTS optomechanics with micrometer screws.

	Element Tolerances
Decenter X	0.01 mm
Decenter Y	0.01 mm
Tilt X	0.01°
Tilt Y	0.01°
